# Improving the quality of obstetric care for women with obstructed labour in the national referral hospital in Uganda: lessons learnt from criteria based audit

**DOI:** 10.1186/s12884-016-0949-1

**Published:** 2016-07-11

**Authors:** Herbert Kayiga, Judith Ajeani, Paul Kiondo, Dan K. Kaye

**Affiliations:** Makerere Univesity College of Health Sciences, Directorate of Obstetrics and Gynecology, P.O.BOX 7072, Kampala, Uganda; Obstetrician/ Gynecologist, Mulago Hospital, P.O.BOX 7051, Kampala, Uganda

**Keywords:** Criteria-based audit, Quality of obstetric care, Obstructed labour, Quality improvement, Uganda

## Abstract

**Background:**

Obstructed labour remains a major cause of maternal morbidity and mortality whose complications can be reduced with improved quality of obstetric care. The objective was to assess whether criteria-based audit improves quality of obstetric care provided to women with obstructed labour in Mulago hospital, Uganda.

**Methods:**

Using criteria-based audit, management of obstructed labour was analyzed prospectively in two audits. Six standards of care were compared. An initial audit of 180 patients was conducted in September/October 2013. The Audit results were shared with key stakeholders. Gaps in patient management were identified and recommendations for improving obstetric care initiated. Six standards of care (intravenous fluids, intravenous antibiotics, monitoring of maternal vital signs, bladder catheterization, delivery within two hours, and blood grouping and cross matching) were implemented. A re-audit of 180 patients with obstructed labour was conducted four months later to evaluate the impact of these recommendations. The results of the two audits were compared. In-depth interviews and focus group discussions were conducted among healthcare providers to identify factors that could have influenced the audit results.

**Results:**

There was improvement in two standards of care (intravenous fluids and intravenous antibiotic administration) 58.9 % vs. 86.1 %; *p* < 0.001 and 21.7 % vs. 50.5 %; *P* < 0.001 respectively after the second audit. There was no improvement in vital sign monitoring, delivery within two hours or blood grouping and cross matching. There was a decline in bladder catheterization (94 % vs. 68.9 %; *p* < 0.001. The overall mean care score in the first and second audits was 55.1 and 48.2 % respectively, *p* = 0.19. Healthcare factors (negative attitude, low numbers, poor team work, low motivation), facility factors (poor supervision, stock-outs of essential supplies, absence of protocols) and patient factors (high patient load, poor compliance to instructions) contributed to poor quality of care.

**Conclusion:**

Introduction of criteria based audit in the management of obstructed labour led to measurable improvements in only two out of six standards of care. The extent to which criteria based audit may improve quality of obstetric care depends on having basic effective healthcare systems in place.

## Background

Obstructed labour is defined as failure of descent of the fetal presenting part in the birth canal in spite of adequate contractions. This is commonly due to cephalo-pelvic disproportion. If not managed promptly, obstructed labour may lead to maternal and fetal complications. Worldwide, obstructed labour occurs in an estimated 5 % of pregnancies and accounts for an estimated 8 % of maternal deaths [1]. In a study conducted in six hospitals in the southwestern part of Uganda [2], the prevalence of obstructed labour was 10.5 %.

Of the 32,511 women who delivered at Mulago hospital, obstructed labour was the main contributor to maternal and fetal morbidity and mortality (Mulago hospital records 2012). For instance, obstructed labour and its complications (uterine rupture, puerperal sepsis, and postpartum hemorrhage) resulted in (40 out of 155) 26 % of all maternal deaths, and led to 266 out of 619 (40 %) admissions to the High Dependence Unit (HDU) (Mulago hospital records 2012). In addition, a review of 70 case files of patients managed for obstetric fistulas in 2012 in the Urogynaecology unit showed that 30 % of the patients had delivered in Mulago hospital following obstructed labour, prior to development of the obstetric fistula. These indicate poor quality of care for obstructed labour.

Poor quality of obstetric care was implicated in approximately 40 % of all maternal deaths in a Nigerian Teaching hospital [3]. There were no local protocols or guidelines for the management of obstructed labour on the obstetric wards in Mulago hospital and clinical management did not depend on pre-determined standards of care. We hypothesized that lack of standardized management protocols for obstructed labour compromised the quality of obstetric care provided to women with obstructed labour, and that the quality of obstetric care could be improved through criteria based audit study. We assumed that when health workers adhered to pre-determined protocols, there would be early recognition of obstructed labour, and that institution of timely interventions could improve the quality of obstetric care [4]. During implementation of the criteria based audit, we developed protocols for clinical management of obstructed labour with the aim of improving the quality of care for women with obstructed labour.

## Methods

### Study setting

This study was conducted in the obstetric wards in Mulago Hospital, Uganda’s National Referral Hospital and teaching hospital for Makerere University College of Health Sciences.

Most of the patients are referred from satellite health facilities around Kampala City. Other patients are referred from Regional referral hospitals over 250 miles from Mulago hospital. Mulago hospital is a government-funded 2700-bed hospital, which currently serves more patients than 60,000 obstetric patients per annum. The hospital has 33,000 deliveries per year. The care is free to the public.

The labour ward receives about 60–80 admissions, delivers about 50–70 mothers by vaginal delivery and 20–25 mothers by emergency caesarean delivery daily in 12-h duty shifts. Most of patients get spinal anesthesia. General anesthesia is only given in cases were spinal anesthesia fails to take or incase of obstetric complications such as HELLP syndrome. Operative vaginal delivery is less than 10 % at this facility.

The study units included the labour ward, postnatal ward, Operating theatre and HDU, which units are adjacent to each other on the same floor of the hospital.

The medical care is provided via a 24-h duty shift team comprised of two specialist obstetricians (as team leaders), seven residents (trainee obstetricians), three Intern doctors, eight midwives, one records officer and three Anesthetic Officers / Anesthesiologist. This translates into provider: patient ratio of 1:5.

### Study design

**Fig. 1:**
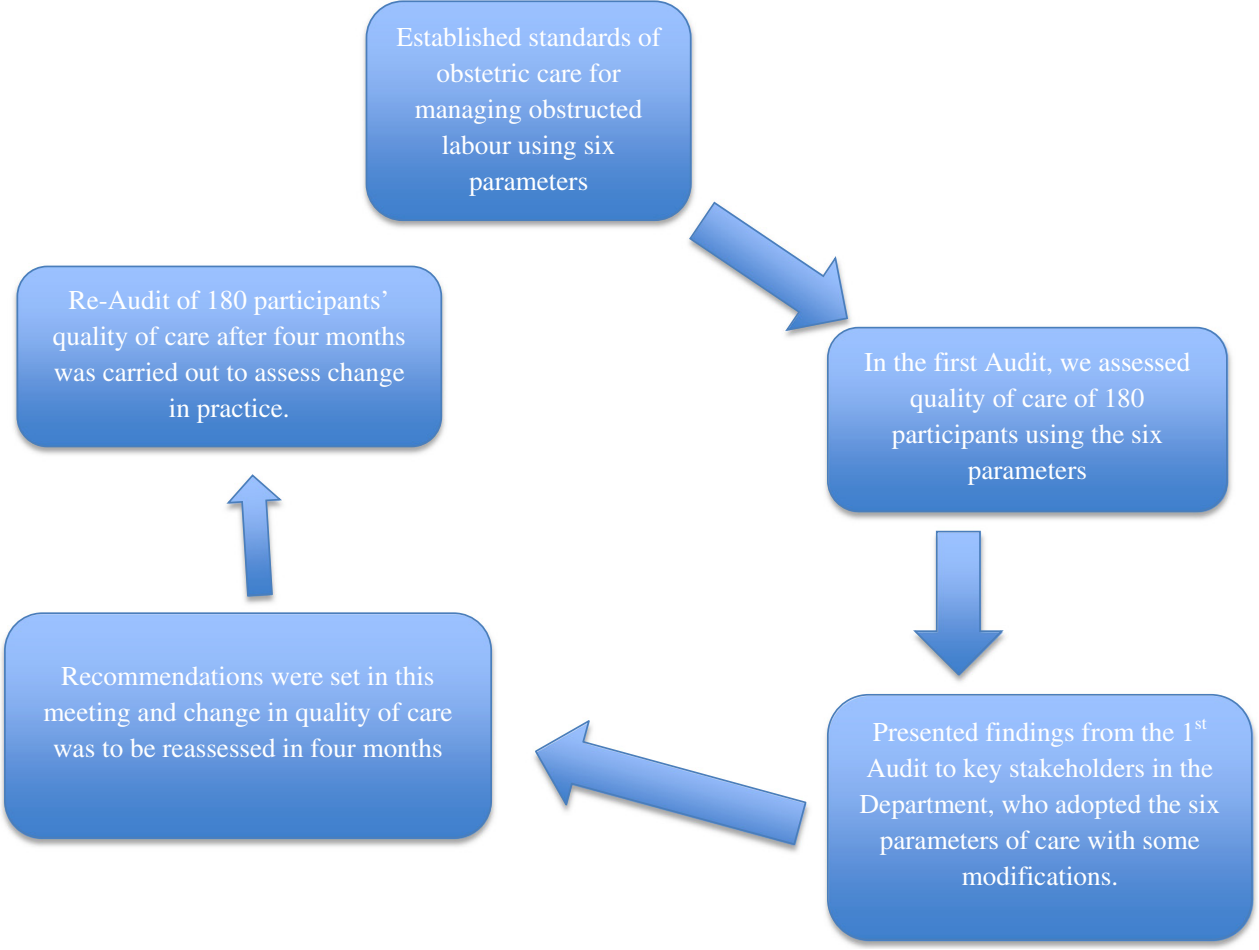
Shows the steps that were followed in the Audit process at Mulago Hospital

The study design was a criteria-based audit conducted prospectively over a period of seven months. Two criteria-based audits were performed, one from September – October 2013 and the second from February – March 2014. In the interim between the two audits, a quality improvement initiative was held in November 2013 when providers and stakeholders were presented with the results from the first audit. The initial audit results were discussed with 53 key stakeholders (specialists, resident obstetricians, intern doctors, midwives and hospital administrators). Gaps in patient management were identified and recommendations for improving obstetric care agreed upon for implementation. Six standards of care (intravenous fluids, intravenous antibiotics, monitoring of maternal vital signs, bladder catheterization, delivery within two hours, and blood grouping and cross matching) were implemented (Tables 1 and 2). Management protocols for obstructed labour including the six standards were developed and implementation was initiated after training of all healthcare providers. Observing criteria-based audit, a review process was conducted where clinicians were to agree on a number of explicit and realistic criteria of good quality for mothers with obstructed labour that were feasible within Mulago hospital [5, 6]. In absence of local protocols and guidelines on management obstructed labour in Mulago Hospital, in this study we used a criteria (standard of care) suggested by Graham et al. with some modifications [7]. A second audit was conducted four months later to evaluate the impact of implementing the recommendations, and results of the two audits were compared and a mean care score computed. In-depth interviews and focus group discussions were conducted to assess factors that could have influenced the results. The steps that were followed in this study are demonstrated in Fig. 1.

**Table 1:** Indicators, their set targets and Action points following presentation of first audit results

Indicator	Currents status (At the initial audit)	Target (By the end of the second audit)	Action points during implementation	Contact Person and level of Achievement of target
Delays	23.3 % had reasons in patient notes for not delivering within the expected 2 h. 85.1 % of the reasons were theatre related	Increase the number of women accessing theatre within two hours from 32 to 64 %.	Scaling up the number of mothers having assisted vaginal delivery. Assigning mothers to midwives for closer observation.	Labour Suite in charge Not achieved
IV fluids assessment prior to delivery	41 % had IV access line 28 % received at least 1 L	100 % for IV access and I litre of IV fluid administration	Better documentation of all IV fluids given. Have a checklist in all patients’ charts	Team on duty. Almost achieved
IV Antibiotic assessment Receiving d IV antibiotics pre-operatively	22 % of mothers.	All mothers to have pre-operative antibiotics	Doctors to prescribe in all patient files	Labour suite weekly rotation Team leader Partly achieved

**Table 2:** Indicators, their set targets and Action points following presentation of first audit results

Indicator	Currents status (At the initial audit)	Target (By the end of the second audit)	Action points during implementation	Contact Person and level of Achievement of target
Ω Blood grouping and cross matching for all patients prior to caesarean delivery	13 % had blood taken off	A target of 100 % was set.	Blood group & CBC were to be sent on.	Labour suite weekly rotation Team leader Target not achieved.
Bladder Catheterization Mothers to have urinary catheters inserted before delivery.	94 % of the mothers Preoperatively	100 % bladder catheterization pre-operatively.	Avail sundries. Avail Obstructed labour management protocol	Labour suite Area manager & Protocol team Protocol developed
Vital sign monitoring Done both on admission and at least once in 4 h in the labour ward.	15 % had a BP 17 % had their pulse rate measured No participant had a single temperature reading taken	100 % monitoring of vital signs (BP, PR, Temperature).	Midwives to take the vital signs CME was to be conducted The labour ward to be zoned into sections. .	Labour Suite in-charge CMEs conducted (achieved) Other targets not achieved

Ω Labour suite laboratory to process all the samples; Doctors and midwives to take off samples; Grouping and cross matching may be done in antenatal care to reduce delays

### Participants’ recruitment

Upon making a diagnosis of obstructed labour by the obstetric team on duty, patients who consented to participate in the study were followed prospectively to the point of discharge from the facility. The quality of obstetric care given to them was analyzed from their charts. Participants were interviewed for details where there was need for clarity in cases of under or over documentation of clinical findings.

Inclusion criteria:

1. All women with obstructed labour managed in Mulago hospital during the study period that consented to participate in the study.

Exclusion criteria:

1. Women managed for obstructed labour in other facilities but referred to Mulago hospital for further care following delivery.
2. Women with more than one obstetric emergency not related to each other e.g., Eclampsia + Obstructed labour.
3. Very sick women with obstructed labour unable to give informed consent or participate in patient interviews.

The study participants were 180 women with obstructed labour in each audit that was conducted within 24 h of admission. The criteria used for diagnosis of obstructed labour included having three adequate uterine contractions in ten minutes lasting 45–60 s with no cervical progression in two hours or progression less than 1 cm/h or presence of clinical signs of obstructed labour (impending uterine rupture, Bandl’s ring, blood-stained urine, severe caput or molding of fetal head) or clinical signs of shock (hypotension or tachycardia with a pulse of >110/min).

### Maternal outcome variables

The maternal outcomes, which were considered in the study included; hospital stay of the participants, mode of delivery, maternal morbidities like puerperal sepsis, uterine rupture, fistula formation, and whether mother died or was alive at discharge.

### Fetal outcome variables

Fetal outcomes that were considered included; admission to a NICU (special care Unit), rationale for admission to the NICU, and whether the baby died or was alive at discharge from hospital.

### Study procedure and data collection

An initial one-week pilot study of ten participants diagnosed and managed for obstructed labour was conducted to pretest the instruments. After revising the instruments, audit participants were recruited from the labour ward and informed consent obtained prior to enrollment into the study.

In addition, participants’ case files were extracted and the quality of care the participants received audited, to assess the clinical management particularly data related to six standards of care (administration of intravenous fluids, intravenous antibiotics, monitoring of maternal vital signs, bladder catheterization, delivery within two hours, and blood grouping and cross matching). Interviewer-administered questionnaires were used to assess socio-demographic data, referral status and other relevant obstetric history related to the clinical management. Participants were interviewed for details where there was need for clarity in cases of under or over documentation of clinical findings. For each participant, the management received was compared to the recommended practice of the set standard of care.

The study criteria (standard of care) for managing mothers with obstructed labour included; delivery by emergency caesarean delivery, destructive vaginal or assisted vaginal delivery within two hours of making a diagnosis of obstructed labour, intravenous access and at least one litre, Intravenous fluids to be given to correct metabolic derangement before delivery, Intravenous antibiotics to be administrated pre-operatively within one hour before any intervention to relieve the obstruction, blood grouping and cross-matching, monitoring temperature, fetal heart rate, pulse rate, blood pressure in an observation chart at least every four hours and bladder catheterization.

### Focus group discussions and in-depth interviews

To explore healthcare providers’ perceptions of the audit findings, ten semi-structured in-depth interviews (with one specialist obstetrician, one resident, the in-charge of the labour ward, the in-charge of theatre and the in-charge of the postnatal ward) and three focus group discussions (FGDs) (separately with midwives, intern doctors and residents) were conducted after each audit. Each FGD had ten participants and lasted about 40–80 min. The questions were thematically related to the criteria-based audit and were open ended. The interviews and FGDs were transcribed, coded and analyzed by thematic analysis.

### Sample size calculation for the audit

Using Kish Leslie formula (1965) for sample size estimation, a mean care score in the initial P_1_ and second audit P_2_ of 81.7 and 93.5 % respectively (from a study conducted in South-western Nigeria [4], an acceptable error margin M of 5 %, a power of 80 % and using the formula n = (*Τ*^2^[*Ρ*_1_(1 − *Ρ*_1_) + P_2_(1 − *Ρ*_2_)])/ *Μ*^2^ with as the standard value of 1.96, the minimum sample size of 324 participants for both audits was computed.

### Data analysis

Data entry was performed with EPI-DATA 3.1 and analyzed using STATA version 12. Results from initial and second audits were compared using chi square (*χ*^2^) for categorical variables and the Student *t*-test for numerical variables, and odds ratios computed. The level of statistical significance was set at *p* < 0.05. The performance score was computed as the number of participants that received the recommended divided by the total number of participants ×100.

## Results

Table 3 shows the socio-demographic and clinical characteristics of the participants, where there were no significant differences in the characteristics of participants in the two audits. The mean age of the participants in the first audit was 23.9 ± 4.7 years while that of the second audit was 23.5 ± 5.7 years. In both audits, the median age of the participants was 23 years with Inter-quartile range of 7 years (age was not normally distributed). Over 50 % of the participants in both audits were prime gravidas. For all participants, a blood pressure and maternal pulse measurement were made at least once during their stay in labour ward before delivery. None of the participants had a single temperature reading obtained while in the labour suite or on admission at any point during both first and second audits. Only five percent of the participants had partographs plotted despite their presence in the case files.

**Table 3:** Baseline characteristics of 360 participants managed for obstructed labour in Mulago Hospital in two Audits 4 months apart

Variable	Audit 1 (*n* = 180) Number (%)	Re-audit (*n* = 180) Number (%)	*P*-value
Age			
≤ 20 years	46 (25.6)	62 (34.4)	0.27
21–25 years	79 (43.9)	57 (31.7)	
26–30 years	45 (25.0)	41 (22.8)	
31–35 years	5 (2.8)	15 (8.3)	
> 35 years	5 (2.8)	5 (2.8)	
Antenatal records			
Available	50 (27.8)	43 (23.9)	0.09
Not available	130 (72.2)	137 (76.1)	
Gravidity			
1 (PG)	98 (54.4)	98 (55.5)	0.15
2–4	71 (39.4)	67 (37.2)	
> 4	11 (6.2)	15 (8.3)	
Complications in previous pregnancy			
Yes	58 (32.2)	33 (18.3)	
No	24 (12.8)	48 (26.7)	0.32
Not applicable (PG)	98 (55.0)	99 (55.0)	
^a^Complications in previous pregnancy			
Obstructed labour	7 (12.1)	7 (28.0)	
Previous scar	13 (22.4)	12 (48.0)	0.83
Cephalo-pelvic disproportion	20 (34.5)	1 (2.0)	
Abnormal lie	4 (6.8)	0 (0.0)	
Fetal distress	3 (5.2)	0 (0.0)	
Not recorded	11 (19.0)	8 (16.0)	
^a^Outcome of last pregnancy (non PGs)			
Miscarriage	7 (8.5)	11 (14.1)	0.26
Fresh still birth	4 (4.9)	0 (0.0)	
Early neonatal deaths	5 (6.1)	0 (0.0)	
Live baby	66 (80.5)	67 (85.9)	
Fetal heart status at admission			
Present and recorded	71 (39.0)	73 (40.8)	0.06
Not recorded	95 (53.0)	96 (53.6)	
Absent	14 (8.0)	10 (5.6)	
When the diagnosis of O.L was made			
On admission	59 (32.8)	64 (35.6)	
Labour suite	121 (67.2)	116 (64.4)	0.10

^a^Used Fisher’s exact test because of low cell frequencies

Table 4 and 5 show the results of the criteria-based audit of the clinical management before and after implementation of the management protocols for obstructed labour. Measurable improvement occurred in only two out of the six standards of care.

**Table 4:** Comparison of 3 Standards of care that changed between Audit 1 and Audit 2

Variable	Audit 1 N (%)	Audit 2 N (%)	OR	95 % CI	*p*-value
Intravenous access achieved					
Yes	178 (98.9)	174 (96.6)	0.66	0.46–1.00	0.29
No	2 (1.1)	6 (3.4)			
IV fluids given before delivery					
Yes	106 (58.9)	155 (86.1)	4.33	2.58–7.25	**<0.0001**
No	74 (41.1)	25 (13.9)			
How much fluid before delivery?					
< 1 l	89 (84.0)	59 (38.1)	0.11	0.06–0.22	**<0.0001**
≥ 1 l	17 (16.0)	96 (61.9)			
IV fluids given after delivery					
Yes	178 (98.9)	157 (87.2)	0.08	0.02–0.33	**<0.0001**
No	2 (1.1)	23 (12.8)			
How much IV fluid after delivery?					
< 500mls	4 (2.2)	2 (1.3)	0.57	0.10–3.14	0.69
≥ 500mls	176 (97.8)	155 (98.7)			
IV antibiotics administered after diagnosis of O.L?					
Yes	39 (21.7)	91 (50.5)	3.70	2.33–5.85	**<0.0001**
No	141 (78.3)	89 (49.5)			
Catheterization done					
Yes	169 (94.0)	124 (68.9)	0.14	0.07–0.28	**<0.0001**
No	11 (6.0)	56 (31.1)			
Catheter was in situ post-op					
Yes	159 (94.1)	83 (66.7)	0.12	0.06–0.27	**<0.0001**
No	10 (5.9)	41 (33.3)			
How long was the catheter in situ?					
≤ 3 days	71 (44.4)	58 (69.9)	2.87	1.64–5.05	**<0.0001**
More than 3 days	88 (55.6)	25 (30.1)

**Table 5:** Comparison of parameters that did not change between Audit 1 and Audit 2

Variable	Audit 1 N (%)	Audit 2 N (%)	OR	95 % CI	*p*-value
Delivery within 2 h of diagnosis of obstructed labor.					
< 2 h	56 (32.6)	58 (33.1)	1.01	0.81–1.26	0.91
≥ 2 h	116 (67.4)	117 (66.9)			
Grouping and Cross matching done before intervention					
Yes	18 (10.0)	25 (14.0)	1.45	0.76–2.76	0.24
No	162 (90.0)	153 (86.0)			
Maternal blood pressure taken at admission & at least once					
Yes	20 (11.1)	16 (8.9)	0.78	0.39–1.56	0.48
No	160 (88.9)	164 (91.1)			
Partograph used to monitor labor					
Yes	18 (10.0)	11 (6.1)	0.59	0.27–1.28	0.17
No	162 (90.0)	169 (93.9)			

Table 6 shows the maternal and fetal outcomes after both audits. Maternal outcomes improved while fetal outcomes deteriorated.

**Table 6:** Fetal and Maternal outcome parameters of the Participants in the two Audits

Variable	Audit 1	Audit 2	*p*-value
	Number (%)	Number (%)	
Baby admitted in Special care Unit after delivery			
Yes	27 (15.2)	31 (17.2)	0.61
No	149 (84.8)	149 (82.8)	0.61
Reason for admission to Special care Unit			
Birth asphyxia	12 (43.9)	14 (45.1)	0.93
Low APGAR	15 (56.1)	9 (29.0)	**0.04**
Neonatal Sepsis	0 (0.0)	8 (25.9)	**0.02**
Baby alive at discharge			
Yes	153 (85.7)	153 (85.0)	0.85
No	27 (14.3)	27 (15.0)	0.85
If baby not alive, is it any of the following?			
Fresh still birth	12 (43.5)	20 (74.1)	**0.02**
Macerated Still birth	07 (26.1)	4 (14.8)	0.30
Early neonatal death	08 (30.4)	3 (11.1)	0.08
Mode of delivery			
Caesarean Section	172 (95.3)	149 (83.7)	**<0.001**
Vacuum delivery	04 (2.5)	10 (5.6)	0.17
Vaginal delivery	02 (1.1)	10 (6.6)	**0.01**
Destructive delivery	02 (1.1)	09 (5.1)	**0.03**
Maternal Morbidity			
Ruptured uterus	08 (43.3)	2 (11.8)	**0.04**
Sepsis/dehiscence	10 (50.0)	2 (11.8)	**0.02**
Obstetric fistula	01 (6.7)	0 (0.0)	0.31
Post spinal headache	0 (0.0)	13 (76.4)	**<0.001**
Duration of hospital stay			
≤ 3 days	50 (30.1)	69 (38.8)	0.08
4–7 days	106 (61.4)	103 (57.9)	0.50
8–14 days	11 (6.2)	4 (2.2)	0.06
> 14 days	04 (2.3)	2 (1.1)	0.38

### Qualitative study findings

Several issues emerged from the in-depth interviews and focus groups, the following key themes were categorized as; a) healthcare provider, b) health facility and c) patient factors.

#### Healthcare provider factors

While participant recognized that the facility was endowed with enough skills to diagnose and treat obstructed labour, there were several challenges from healthcare providers. These include healthcare providers’ attitude, lack of teamwork, declining commitment to serve, lack of staff supervision, and the lack of task allocation and apportioning blame. Most participants believed that proper management depends more on health workers’ attitudes than their numbers and believed that there was need for attitude change, as exemplified by one resident:

*“We are few but does it need many midwives to measure blood pressure? You can have 4 midwives and yet not a single Blood Pressure reading is taken any patient for the whole day! …another example, a shift may have 25 patients with 4 midwives but no single partograph is plotted.”*

Likewise, the healthcare providers believed there was a need for attitude change to desist from considering obstructed labour as a “lesser emergency”, in comparison to other conditions like postpartum hemorrhage and birth asphyxia. Participants reported that obstructed labour was given less attention or priority (as evidenced by not monitoring vital signs and poor documentation) and this affected the quality of care provided, as noted by a specialist:

“*At times there is one BP machine, I need to walk around and look for it but another clinician with a poor attitude will not be bothered. It is challenging but we have to work with the few resources available, so that attitude change is needed.”*

Indeed some healthcare providers believed in having a passion and self-sacrifice while treating patients, in spite of the working environment, was necessary in patient care as noted by a theatre staff:

*“Patients don’t receive drugs because no one seems to care. There was a patient who had a fresh stillbirth following obstructed labour and she had a C-section and was taken to postnatal ward. When I found this patient had not taken a shower for a while and did not receive antibiotics just because she had no IV access [cannula]. I bathed her and gave the drugs myself”.*

Since a multidisciplinary team involving a doctor, midwife and anesthetist best manages obstructed labour, participants were concerned that this did not happen very often because many times one of them, especially the Anesthetist or doctor, would be unavailable. In addition, midwives and doctors appeared to work independently and not as a team, which led to communication gaps between them, particularly where there were hierarchical differences, as noted by a midwife:

*“Doctors are too proud especially when called upon to review some mothers with complications, which discourages us from consulting them, leading to delays… Both nurse midwives and doctors do separate rounds instead of working together. There is an ‘I know it all!’ mentality”.*

However, some healthcare providers thought teamwork is not always possible, that with the many patients, they have to separate to cover as many patients as possible in the shortest possible time. In addition, junior doctors have to run errands such as looking for blood, thereby missing ward rounds, as noted by a specialist:

*“There is some teamwork but it’s difficult, and apparently teamwork may not seem to work. When we have a doctor, midwife, and an intern, we can’t move as a team to do the work, as we may need to separate to do the work promptly. We allow the team to disintegrate but we keep communicating to each other.”*

Participants reported declining healthcare provider commitment to work, absenteeism and low morale due to high patient load, poor remuneration and frustrations from inadequate supplies, as noted by a labour ward staff:

*“There are constant stock outs of supplies, the pay is so low yet the patients are too many. … I am disgusted with the whole system and I feel like leaving. The work is constantly frustrating and I’m no longer excited about work.”*

#### Health facility factors

Lack of task allocation and apportioning blame were identified as contributory to poor quality of care. Poor monitoring of patients was partly attributed to lack of allocation of tasks, and each party thinking the other will handle the case, and when the task remains undone, everyone blames the other. Where tasks are delineated, inadequate supervision for intern doctors, residents, medical students and midwives was perceived as a factor that contributes to poor quality of care, and yet numbers of these have progressively increased, as noted by a resident:

*“There are supervision issues as there are few specialists who show up on the wards to provide guidance so we can better manage the patients.”*

Stock-out of essential supplies, drugs, equipment and sundries was identified as a significant contributor poor management of obstructed labour, as noted by a labour ward staff:

*“As the financial year closes we suffer marked shortage of supplies because of the delay of release of funds, and the situation has kept worsening over time. On a typical day we do 20-25 Caesarean sections, but when I requisition for Foley’s catheters for the week, I am given 30 to cover a week, which can only last one day.”*

Poor documentation was attributed to be to occasional absence of appropriate stationery, as noted by an intern doctor. This may affect regular use of a partograph:

*“Sometimes documentation is less because there is lack of appropriate stationery in patient files. For example, when you have no partograph, how do you plot?”*

The labour ward theatre itself had challenges. To improve management of obstructed labour, you need a theatre that is readily accessible when needed so as to minimize delays in providing timely intervention. Frequent stock-outs of theatre supplies like linen, and shortage of anesthetists contribute to accumulation of caesarean section caseload leading to delays. Such delays and stock-outs contribute to poor quality of care for obstructed labour in spite of recommended guidelines, as noted by a labour suite staff.

*“In the theatre, one team member may be unavailable. Some members may be there, but when another comes and finds one (key) person is missing on the team, they also disappear!*

Absence of management protocols at lower health units was singled out as a challenge, leading to inappropriate or delayed referrals. The healthcare providers there used old protocols in the midst of changing guidelines, as noted by an intern doctor:

*“The challenge starts with the lower health centres as most of the mothers are referrals; the people make a wrong diagnosis and usually refer to it as prolonged labour or delay in second stage, making it difficult to manage in time. The system does not separate levels of complications and emergencies.”*

Participants reported that with the lack of resources at the facility, patients are often expected to buy materials such as drugs. However, many patients do not have money to afford these requirements, which compromises the quality of care provided to the patients, as noted by a postnatal ward staff.

*“Most of the time, we encounter problems like the drugs are out of stock. We tell them to buy but they have no money delaying and compromising the care given, and this causes other complications like ruptured uterus and sepsis.”*

#### Patient factors

During the interviews and FGDs, it was emphasized that there was a high patient load attended to by few staff, particularly over the weekend other health facilities are closed. The high caseload exhausts available resources. The midwife to mother ratio did not offer a favorable environment for adequate obstructed labour management as expressed by a labour ward midwife:

*“Nurses are the ones who stay on the bedside 24 hours yet the patients are too many for these nurses to handle adequately. (To manage obstructed labour, you need a midwife to mother ratio of 1:3 but we receive a tune of up to 100 patients in a day and out of that number we deliver about 80 per day for both spontaneous vaginal delivery and caesarean section. This is a high number compared to the midwives who are about 2-3 in the delivery suite. This makes it hard to do key vital sign monitoring for the patients*.”

Patient delay in seeking appropriate medical care was perceived to contribute to poor outcomes of obstructed labour. Mothers seek medical care when obstructed labour is advanced, as noted by an intern doctor:

*“Mothers first try to seek help elsewhere till they are referred to us. They come in late when there are other priority cases already on the theatre list; yet each delay worsens the situation.”*

Patients need to be taught to respect medical advice and comply with instructions. Some patients decline or deviate from post-operative protocols, as reported by a postnatal midwife:

*“The patient also contributes. Some have a negative attitude on bladder catheterization. Some pressurize medical workers or pay money for the catheters to be removed before the scheduled removal time.”*

## Discussion

This study aimed at assessing extent to which criteria-based audit improves the quality of obstetric care given to women with obstructed labour. The results show that introduction of criteria-based audit in the management of obstructed labour led to measurable improvements in only two out of six standards of care. Healthcare factors, facility factors and patient factors contributed to failure of quality improvement on the recommended standard of care. Though there was a decline in the overall mean performance score from 55.1 % in audit one to 48.2 % in second audit, there was a slight improvement in some parameters assessed in the second audit.

The value of criteria-based clinical audit in improving quality of obstetric care could be assessed objectively in terms of the change in the proportion of cases where management met the criteria for good quality care [8]. There is no stipulated duration of time that must be observed between the two audits. In Weeks et al. audit study [5], improvements were seen over a period of six months. Crombie, however, noted that effecting quality takes time; thereby emphasizing that even the most likely outcome of a well conducted audit study is only partial success [9]. In this study, there were limited funds to allow a wider time interval between the first and second audit. Given more time, and if provider and facility concerns had been addressed, the results of the second audit could have been different.

The findings show that improvement in quality of obstetric care without strengthening health systems may have minimal success. In the midst of scarcity of resources in sub-Saharan countries, Kidanto et al. [10] found that efforts to improve maternal and newborn quality of care were self-driven without mobilizing external resources. This called for reorganization of available human resources (physicians and nurses) in all levels accompanied by self-motivation to combat the unacceptably high maternal and child morbidity and mortality in the Sub-Saharan countries [10]. Efforts to improve the outcomes of the second audit like re-organization of the labour ward (like supervision, team work, motivation and task allocation) to ensure maximum utilization of the available human resources were not implemented. This could explain the poor results of the second audit.

The study findings demonstrate constraints and challenges that may hinder quality improvement through criteria-based audit. Of the constraints described by Ronsmans [6] for successful implementation of audits in low resource countries, this study faced challenges of low motivation from the health care providers, short time between the first and second audits because of limitations in the study funding, poor documentation of clinical findings in patients’ case files, stock outs of medical sundries and equipment and the short duration residents and intern doctors rotate in the labour ward. The latter necessitates regular training and updating on management protocols.

Ten very sick patients were excluded because they were too sick to consent and also participate in the interview. They also had other co-morbidities that could have altered the study findings.

Being a national referral and teaching hospital, the intern doctors and residents (trainee obstetricians) rotate in the labour ward for one week every one to two months, thereafter shifting to other wards. The specialist obstetricians/gynecologists rotate in the labour ward once every fortnight. These changes in the staff make it hard to ensure supervision or maintain consistent quality of care in the labour ward.

As in other studies [11], compliance to the patient management protocol was still low for all cadres. Most of the parameters that did not change in the second audit seemed to depend more on health systems strengthening efforts than the individual staff efforts. Such challenges are not unique to this study alone, and have been reported by other researchers [5, 6, 12–14].

Amidst all the constraints, in this study it has been found that promotion of the audit cycle is feasible and a useful strategy to improve professional practice in under-resourced settings where adherence to recommended practice is low [15]. This because as health care providers come together to solve health problems as part of the audit cycle, they can formulate practical solutions that may improve their patient care within their setting thereby enriching and improving their professional practice.

## Conclusion

The introduction of criteria-based audit in the management of obstructed labour was well received and led to measurable improvement in two standards of care provided to the study participants, though overall improvement in practice was minimal. Putting management guidelines and protocols in place to streamline patient management, without having a well-motivated team and constant supply of materials, may be inadequate to improve patient care. The extent to which criteria-based audit may improve the quality of care provided to patients is therefore dependent on having effective health systems even in low resource settings.

## Abbreviations

BP, Blood pressure; CME, Continuing Medical Education; C-section, Caesarean section; FGDs, Focus Group Discussions; HDU, High Dependency Unit; HELLP syndrome, Hemolytic Anemia Elevated liver enzymes and Low Platelets; IV, Intravenous; NICU, Neonatal (Special care) Intensive Unit; OL, Obstructed labour; PR, Pulse Rate

## References

[1] Adhikari S, Dasgupta M, Sanghamita M (2005). Management of obstructed labour: a retrospective study. J Obstet Gynecol India.

[2] Kabakyenga JK, PerOlof Ö, Eleanor T, Mukasa PK, Pettersson KO (2011). Individual and health facility factors and the risk for obstructed labour and its adverse outcomes in south-western Uganda. BMC Pregnancy Childbirth.

[3] Okonofua FE, Abejide A, Roger A, Makanjuola RA (1992). Maternal mortality in Ile-Ife, Nigeria: a study of risk factors. Stud Fam Plan.

[4] Hunyinbo K, Fawole A, Sotiloye O, Otolorin E (2008). Evaluation of criteria-based clinical audit in improving quality of obstetric care in a developing Country hospital. Afr J Reprod Health.

[5] Weeks AD, Alia G, Ononge S, Otolorin E, Mirembe FM (2005). A criteria-based audit of the management of severe pre-eclampsia in Kampala, Uganda. Int J Gynecol Obstet.

[6] Ronsmans C (2001). What is the evidence for the role of audits to improve the quality of obstetric care?. Stud Health Serv Organ Policy.

[7] Graham W, Wagaarachchi P, Penny G, MacCaw-Binns A, Yeboah A, Hall M (2000). Criteria for clinical audit of the quality of hospital based obstetric care in developing countries. Bull WHO.

[8] Wood L (2002). Making audit work. Obstet/Gynecol.

[9] Crombie I, Davies H, Abraham S, FCdV (1997). The audit handbook. Improving health care through audit.

[10] Kidanto HL, Peter W, Charles DK, Lennarth N, Gunnila L (2012). Improved quality of management of eclampsia patients through criteria based audit at Muhimbili National Hospital, Dar es SalaamTanzania. Bridging the quality gap. BMC Pregnancy and Childbirth.

[11] Tuffnell D, Jankowicz D, Lindow S, Lyons G, Mason G, Russell I (2005). Outcomes of severe pre-eclampsia/eclampsia in Yorkshire 1999/2003. BJOG.

[12] Jamtvedt G, Young JM, Kristoffersen DT, O’Brien MA, Oxman AD. Audit and feedback: effects on professional practice and health care outcomes. Cochrane Database Syst Rev. 2006;2(2):10–13. 10.1002/14651858.CD000259.pub2 16625533

[13] Wagaarachchi PT, Fernando L (2002). Trends in maternal mortality and assessment of substandard care in a tertiary care hospital. Eur J Obstet Gynecol Reprod Biol.

[14] Liabsuetrakul T, Peeyananjarassire K, Tassee S, Sanguanchua S, Chaipinitpan S (2007). Emergency obstetric care in the southernmost provinces of Thailand. Int J Qual Health Care.

[15] Wagaarachchi PT, Graham WJ, Penney GC, McCaw-Binns A, Antwi KY, Hall MH. Holding up a mirror: changing obstetric practice through criterion-based clinical audit in developing countries. International Journal of Gynecology & Obstetrics. 2001;74(2):119–130. 10.1016/s0020-7292(01)00427-1 11502289

